# *In utero* Exposure to Atrazine Disrupts Rat Fetal Testis Development

**DOI:** 10.3389/fphar.2018.01391

**Published:** 2018-11-28

**Authors:** Yinghui Fang, Chaobo Ni, Yaoyao Dong, Huitao Li, Siwen Wu, Xiaoheng Li, Yao Lv, Tongliang Huang, Qingquan Lian, Ren-shan Ge

**Affiliations:** ^1^Department of Anesthesiology, Perioperative and Pain Medicine, Key Laboratory of Anesthesiology of Zhejiang Province, Zhejiang, China; ^2^Department of Pharmacy, The Second Affiliated Hospital and Yuying Children’s Hospital, Wenzhou Medical University, Wenzhou, China

**Keywords:** atrazine, fetal Leydig cell, leydig cell aggregation, sertoli cell, testosterone

## Abstract

Atrazine (ATR) is a commonly used agricultural herbicide and a potential endocrine disruptor that may cause testicular dysgenesis. The objective of the present study was to investigate the effects of atrazine on fetal testis development after *in utero* exposure. Female Sprague-Dawley rats were gavaged daily with vehicle (corn oil, control) or atrazine (25, 50, and 100 mg/kg body weight/day) from gestational day 12 to 21. Atrazine dose-dependently decreased serum testosterone levels of male pups, with a significant difference from the control recorded at a dose of 100 mg/kg. In addition, atrazine significantly increased fetal Leydig cell aggregation at a dose of 100 mg/kg. Atrazine increased fetal Leydig cell number but not Sertoli cell number. However, atrazine down-regulated *Scarb1* and *Cyp17a1* in the fetal Leydig cell *per se* and *Hsd17b3* and *Dhh* in the Sertoli cell *per se*. These results demonstrated that *in utero* exposure to atrazine disrupted rat fetal testis development.

## Introduction

A cluster of clinical manifestations, including the male reproductive tract malformation (cryptorchidism and hypospadias), testicular cancer, infertility, and subfertility, is referred as the testicular dysgenesis syndrome (Skakkebaek et al., 2001). Although the exact causes are not known, testicular dysgenesis syndrome is believed to be caused by the intrauterine factors, including genetics and exposure to chemicals (Skakkebaek et al., 2001). Indeed, some anti-androgens, such as phthalates and methoxychlor, can produce such manifestations in rodent models (Fisher et al., 2003; Hu et al., 2009; Liu et al., 2016), resulting in the hypothesis that testicular dysgenesis syndrome is caused by the environmental endocrine disruptors, which get into the fetal circulation via maternal circulation.

One such endocrine disruptors could be atrazine (ATR, 6-chloro-N^2^-ethyl-N^4^-isopropyl-1, 3, 5-triazine-2, 4-diamine). ATR was first introduced to the market as an herbicide in 1957. ATR belongs to the family of 6-chloro-s-triazine herbicides, which also include other members, cyanazine, propazine, and simazine. ATR is used to control a wide spectrum of broadleaf weeds and grasses primarily around crops (Bridges et al., 2008). It is the most widely used herbicide globally. The chemical structure of ATR is stable and has low chemical reactivity. Therefore, ATR persists in the soil after long-term application (Frank and Sirons, 1985), resulting in its accumulation in ground and surface water (Ritter, 1990). Residual amounts of ATR and its metabolites have been found in drinking water and agricultural food products (Balinova, 1993; Molinari et al., 1995). ATR, after absorbed, mainly distributes into the liver and kidney (Hanioka et al., 1998).

Increasing evidence shows that ATR is a potential endocrine disruptor. ATR disrupts reproductive function in adult and peripubertal male and female rodents (Eldridge et al., 1999; Trentacoste et al., 2001; Friedmann, 2002; Cooper et al., 2007; Victor-Costa et al., 2010). Although one study demonstrated that ATR also goes through the placenta to enter the fetal circulation to inhibit testosterone production in fetal testis of males, the exact mechanism is still unclear (Fraites et al., 2011). Here, we report that gestational exposure to ATR causes profound leisure of fetal Leydig and Sertoli cells.

## Materials and Methods

### Animals and Materials

Atrazine was purchased from Sigma (St. Louis, MO, United States). SYBR Green qPCR Kit and Protein Assay Kit were purchased from Takara (Otsu, Japan). Trizol Kit was purchased from Invitrogen (Carlsbad, CA, United States). Immulite2000 Total Testosterone Kit was purchased from Sinopharm (Hangzhou, China). Antibody information was presented in Supplementary Table S1. Adult male and female Sprague-Dawley rats were purchased from Shanghai Laboratory Animal Center (Shanghai, China). The experimental protocol for animal toxicity study was approved by Wenzhou Medical University Laboratory Animal Ethics Committee and in conformity with procedures described in the Guide for the Care and Use of Laboratory Animals published by NIH (United States). The detailed methods are listed in Supplementary Data Sheet S1.

### Animal Mating

Rats were allowed one week to acclimate. One female rat and one male rat were randomly picked and mated. Each dam was housed individually in an individual ventilated cage at the room with the following conditions (temperature: 23 ± 2°C, relative humidity: 55 ± 5%, light-dark cycle: 12 h).

### Animal Treatment

Dams were randomly divided into 4 groups with 6 rats per group. ATR was dissolved in corn oil (vehicle control). Each dam was daily gavaged with 1 ml/kg vehicle at control group or 1 ml/kg ATR at doses of 25, 50, or 100 mg/kg body weight from GD12 to GD21. Dose range was selected based on a previous reproductive toxicity study (Desesso et al., 2014), in which ATR doses were within a range of 1–125 mg/kg. The body weights of dams were recorded daily. Dams were allowed to give birth at GD21. Dams were euthanized by CO_2_ at GD21. We measured body weights of dams and male fetuses. The average of body weight of male fetuses from each dam was used as one sample size. We collected serum samples by centrifugation of blood samples of male fetuses. Serum samples from male fetuses of the same dam were pooled together. One set of fetal testes (at least one testis per male fetus per dam) was randomly selected and frozen in liquid nitrogen and stored at -80°C for subsequent real-time quantitative PCR (qPCR) measurement of fetal testicular mRNA levels and Western blot measurement of the levels of some selected testis proteins. Another set of testes (at least one testis per male fetus per dam) was randomly selected and fixed by immersing them in Bouin’s solution for one day for histochemical, immunohistochemical, and immunofluorescent stainings.

### Serum Testosterone Assay

We measured serum testosterone levels by immunochemiluminometric assay using the IMMULITE^®^ 2000 Immunoassay Kit (Sinopharm, Hangzhou, China) as previously described (Li et al., 2018). The minimal detection limit of testosterone was 0.2 ng per mL.

### Hematoxylin and Eosin (H&E) Staining

Some endocrine disruptors induce the increased incidence of multinucleated gonocytes (MNGs) in the fetal testis during the gestational exposure (Borch et al., 2006; Li et al., 2014). Bouin’s solution fixed testes were arranged in a tissue-array in one paraffin block. Cross sections (6 μm) were performed and stained with H&E staining solution. Images of cross sections of the fetal testis were taken. We analyzed one complete testis cross-section of each male fetal rat and enumerated the number of the seminiferous cord in the cross-section that contains MNGs and calculated its percentage of the total tubules counted.

### Immunohistochemical Staining and Cell Counting

Cholesterol side-chain cleavage enzyme (CYP11A1) is the first enzyme that appears in fetal Leydig cell lineage (Ye et al., 2017) and serves as the biomarker for fetal Leydig cells. SOX9 is the first transcription factor that appears in fetal Sertoli cell lineage and serves as the biomarker of fetal Sertoli cells (Koopman, 1999). The numbers of CYP11A1-positive fetal Leydig cells and SOX9-positive fetal Sertoli cells were counted using a fractionator technique as previously described (Mendis-Handagama et al., 1989). In brief, six testes per group were embedded in paraffin in a tissue array. Paraffin blocks were sectioned in 6 μm-thick sections. About ten sections were randomly sampled from each testis per male fetus. Sections were used for immunohistochemical staining. Using the live image of a digital camera, under a 10 × objective, and starting at a fixed point of the “upper” sections, total microscopic fields per section were counted. The total number of Leydig or Sertoli cells was calculated by multiplying the number of Leydig or Sertoli cells counted in a known fraction of the testis by the inverse of the sampling probability.

### Quantitative Immunohistochemical Measurement of CYP11A1 and SOX9

A protein level in the tissue not only relies on its expression level but also on the cell number. The levels of CYP11A1 per fetal Leydig cell and SOX9 per fetal Sertoli cell were measured using quantitative immunohistochemical staining. The density of the target protein (CYP11A1 and SOX9) and background area nearby was measured using the Image-Pro 6 Plus software (Media Cybernetics, Silver Spring, MD, United States) (Liu et al., 2016). Sixty fetal Leydig or Sertoli cells per testis were calculated and averaged for one sample size per group as previously described (Liu et al., 2016).

### Measurement of Leydig Cell Proliferation

The proliferation of fetal Leydig cells was judged by immunofluorescent staining of PCNA after the dual staining of PCNA (a biomarker for proliferating cell) and CYP11A1 (a biomarker for fetal Leydig cell) in the testis. We assembled the sections of fetal testis in a tissue-array as above. We incubated the sections with the primary antibodies of CYP11A1 and PCNA sequentially for 60 min. We then used the fluorescent secondary antibody (Alexa-conjugated anti-rabbit or anti-mouse IgG, 1:500) to label fetal Leydig cell (CYP11A1, mitochondrial staining in green color) and proliferating cell (PCNA, nuclear staining in red color). Images were taken with a fluorescent microscopy and merged.

### Real-Time Quantitative PCR (qPCR)

To determine Leydig and Sertoli cell gene expression levels, we used a qPCR method. We extracted total RNA from a frozen testis using TRIzol. We synthesized the first strand cDNA from total RNA and performed qPCR as previously described. We used ribosomal protein S16 (*Rps16*) mRNA level in each sample as the internal control as previously described (Liu et al., 2016). Primers for testicular genes of interest are listed in Supplementary Table S2, including Leydig cell genes, luteinizing hormone receptor (*Lhcgr*), cholesterol high density lipoprotein receptor (*Scarb1*), steroidogenic acute regulatory protein (*Star*), CYP11A1 (*Cyp11a1*), HSD3B1 (*Hsd3b1*), CYP17A1 (*Cyp17a1*), HSD17B3 (*Hsd17b3*), and INSL3 (*Insl3*), as well as Sertoli cell genes, FSHR (*Fshr*), DHH (*Dhh*), AMH (*Amh*), and SOX9 (*Sox9*). The relative mRNA levels of the target genes were normalized to *Rps16* ( a house-keeping gene) using a standard curve method as previously described (Ge et al., 2005). Because mRNA levels in the testis are influenced by cell number, we adjusted the mRNA levels by either Leydig cell number or Sertoli cell number as the following formulae: adjusted mRNA level = original mRNA level/cell number per testis in the control group.

### Western Blot

We homogenized fetal testes and lysed them using a radio-immunoprecipitation assay buffer (Bocai Biotechnology, China). We determined total protein concentrations using the BCA assay kit according to the manufacturer’s instruction (Galen Biopharm, Beijing, China). Protein in the amount of 30 g for each sample was loaded to a SDS–PAGE gel (10% w/v acrylamide) and electrophoresed and then blotted onto a polyvinylidene fluoride membrane (Bio-Rad, Hercules, CA, United States). Nonspecific bindings were blocked with nonfat milk powder (5% w/v) in a tris-buffered saline tween-20 buffer (TBST) for 1 h. After that, the membranes were incubated at 4°C overnight with primary antibodies against the antigens (listed in the Supplementary Table S1). The membranes were then washed and incubated with HRP-conjugated anti-rabbit or anti-goat IgG secondary antibody (1:2000, Abcam, San Francisco, CA, United States) for 2 h at room temperature and washed 3 times. Blots were stripped and incubated with a polyclonal-actin (ACTB) antibody (as the internal control). The band was visualized and the density was calculated using J-Software.

### Statistical Analysis

All data are presented as the mean ± standard errors (SE) and data are analyzed by one-way ANOVA and then *ad hoc* Dunnett’s test to compare value from ATR-treated groups with the control. GraphPad Prism (Version 6 GraphPad Software, San Diego, CA, United States) was used. *P* < 0.05 is regarded as a significant difference between two groups.

## Results

### General Parameters of Reproductive Toxicology

**FIGURE 1:**
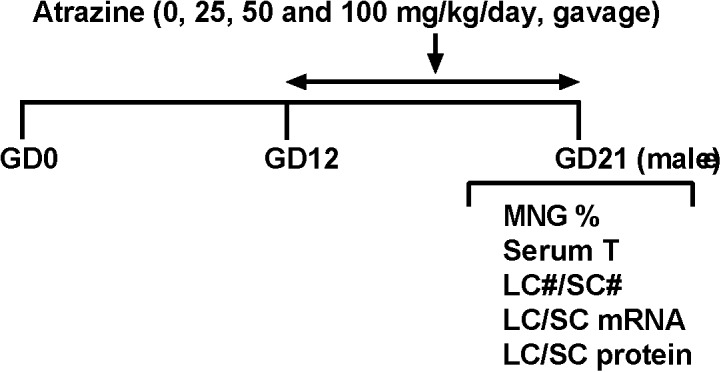
Regimen of atrazine. Dams were gavaged atrazine (0, 25, 50, and 100 mg/kg) from gestational day (GD) 12 to 21. Serum T, serum testosterone level; LC#, Leydig cell number; SC#, Sertoli cell number; MNG% means the occurrence rate of multinucleated gonocytes.

Dams were gavaged 0, 25, 50, and 100 mg/kg/day ATR from GD12 to GD21 (Figure 1). Body weights of dams before and 10 day after 25–100 mg/kg ATR treatment did not change (Table 1). Birth rate, pup number per dam, and percent male pup ratio did not change after ATR treatment, suggesting that ATR does not cause abortion and the change of male to the female sex ratio of pups. Male birth weights after ATR were not different from the control, suggesting that ATR does not cause intrauterine growth retardation. No morbidity and mortality of dams and fetuses were found.

**Table 1:** General reproductive parameters in rats in utero exposed to Atrazine (ATR).

	ATR (mg/kg daily)			
	
	0	25	50	100
**Dams**				
Number of dams	6	6	6	6
Body weight of GD 12 (g)	297.5 ± 9.684	293.2 ± 5.578	289.8 ± 5.545	294.5 ± 9.627
Body weight 10 days after treatment (g)	379.1 ± 6.463	372.6 ± 6.099	371.2 ± 8.403	382.5 ± 9.930
Pup number	73	82	87	77
Pup number per dam	12 ± 1	14 ± 1	14 ± 1	13 ± 1
Birth rate	6/6	6/6	6/6	6/6
Pup male, %	46 ± 5	51 ± 4	57 ± 5	50 ± 7
**Male pups**				
Number of pups	32	43	49	38
Body weight	6.91 ± 0.21	6.76 ± 0.24	6.33 ± 0.15	6.53 ± 0.10


*Dams of Sprague Dawley rats were gavaged with ATR from GD12-GD21. Values are mean ± SEM, *n* = 6. No significant difference between two groups was observed.*

### ATR Does Not Induce MNGs

Some endocrine disruptors, including phthalates, can cause the increase in the incidence of MNGs in the fetal testis (Mahood et al., 2007). The occurrence rate of MNGs after ATR treatment was counted. As shown in Supplementary Figure S1, ATR did not increase MNG incidence when compared to the control. This suggests that ATR does not have a similar toxicity mechanism to the phthalates.

### ATR Decreases Serum Testosterone Levels of Male Pups

**FIGURE 2:**
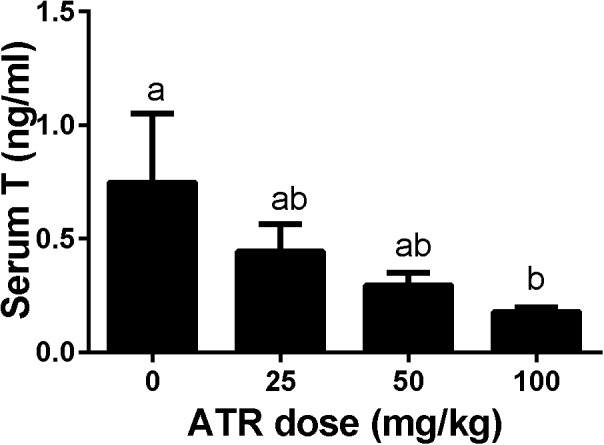
Atrazine decreases serum testosterone levels of male fetuses. The serum testosterone levels after atrazine (ATR) treatment was measured. Mean ± SE, n = 6. Identical letters designate no difference between two groups at P < 0.05.

Dams were gavaged ATR (0–100 mg/kg) from GD12 to GD21, and male pups were analyzed. Serum testosterone concentrations were measured. As shown in Figure 2, ATR significantly decreased serum testosterone levels at a dose of 100 mg/kg. This indicates that ATR suppresses androgen production.

### ATR Increases the Number of Fetal Leydig Cells

**FIGURE 3:**
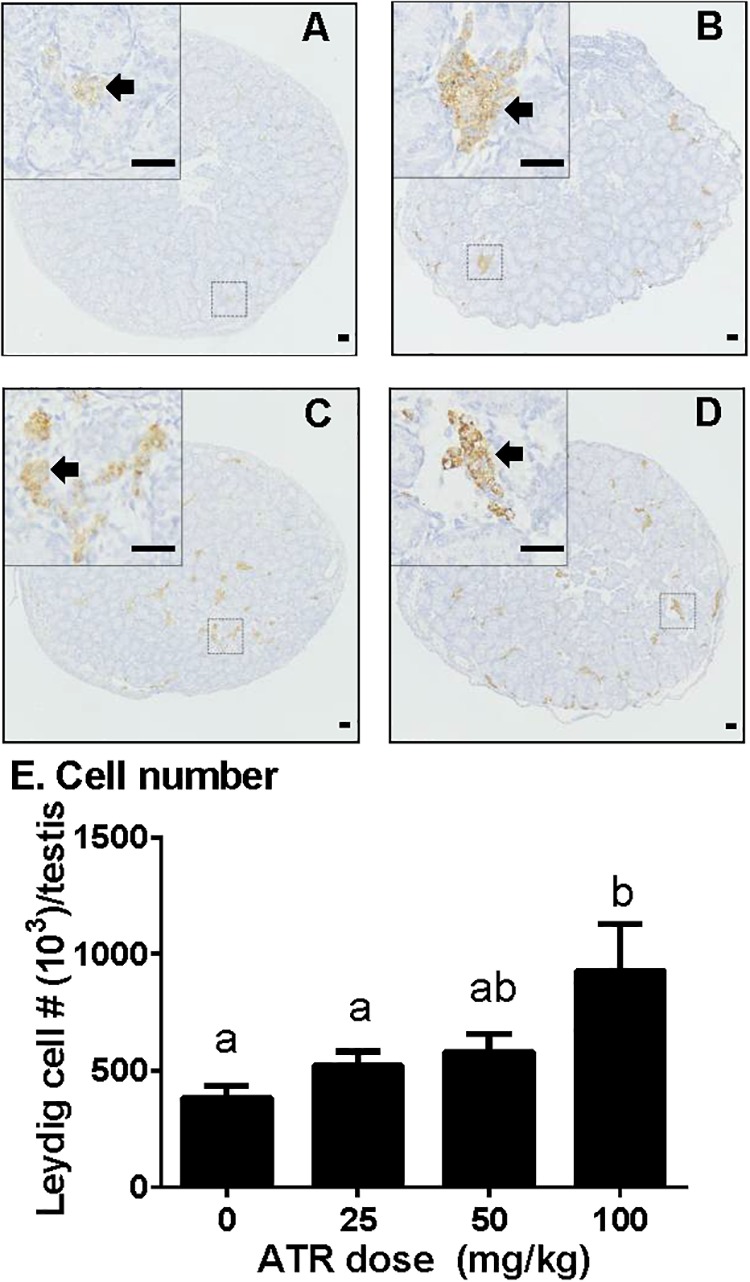
Atrazine increases the number of fetal Leydig cells. Sections were stained immunohistochemically for CYP11A1. The representative photomicrographs of rat testis sections after the treatment of 0 **(A)**, 25 **(B)**, 50 **(C)**, and 100 mg/kg **(D)** ATR. Black arrows point to fetal Leydig cells in the testis. Scale bar = 50 μm. **(E)** Quantitative data of fetal Leydig cell number. Mean ± SE, *n* = 6. Identical letters designate no difference between two groups at *P* < 0.05.

Fetal Leydig cells were labeled by CYP11A1 and enumerated. As shown in Figure 3, ATR significantly increased the number of fetal Leydig cells per testis at a dose of 100 mg/kg. This indicates that ATR affects fetal Leydig cell development.

### ATR Increases Fetal Leydig Cell Proliferation

**FIGURE 4:**
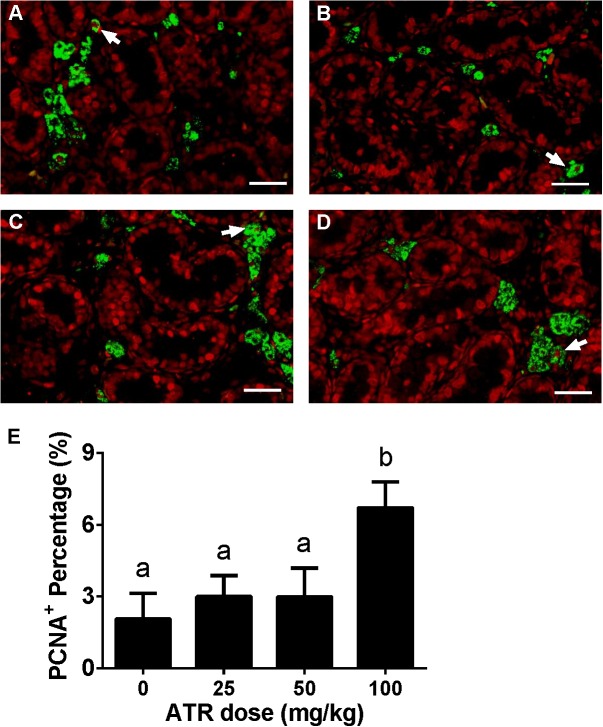
Effects of atrazine on fetal Leydig cell proliferation. Sections were dually stained with PCNA (the biomarker for a proliferating cell) and CYP11A1 (the biomarker for the fetal Leydig cell). Proliferation Leydig cell nuclear was labeled by red color, and all Leydig cell was labeled by green color. The representative photomicrographs of rat testis sections after the treatment of 0 **(A)**, 25 **(B)**, 50 **(C)**, and 100 mg/kg **(D)** ATR. White arrows point to PCNA-positive/CYP11A1-positive cells. Scale bar = 50 μm. **(E)** The percentage of PCNA-positive/CYP11A1-positive cells of total fetal Leydig cells. Mean ± SE, *n* = 6. Identical letters designate no difference between two groups at *P* < 0.05.

Fetal Leydig cell proliferation was labeled by dual stainings of CYP11A1 (a Leydig cell biomarker) and PCNA (a proliferating cell biomarker). As shown in Figure 4, ATR increases the PCNA-labeling index of fetal Leydig cells at a dose of 100 mg/kg. This indicates that ATR increases fetal Leydig cell number via its mitosis.

### ATR Induces Abnormal Aggregation of Fetal Leydig Cells

**FIGURE 5:**
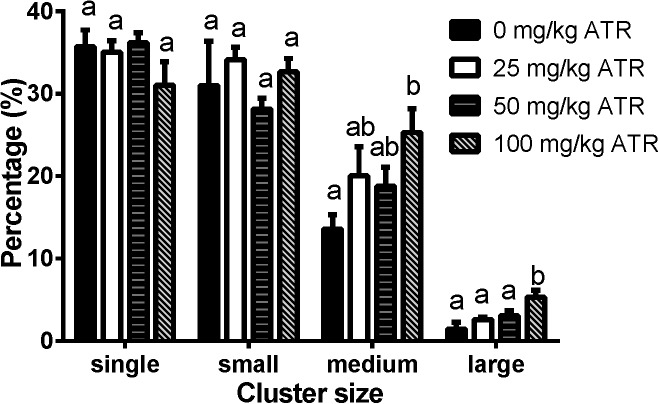
Atrazine induces abnormal aggregation of fetal Leydig cells. Fetal Leydig cells were labeled by CYP11A1 and the percentage of fetal Leydig cell cluster: single, small, medium, and large represent 1, 2–4, 5–16, and > 16 fetal Leydig cells per cluster. Mean ± SE, *n* = 6. Identical letters designate no difference between two groups at *P* < 0.05.

Fetal Leydig cells are distributed as single cell or clusters with 2 or more cells in the interstitium of the testis. In the present study, we defined the cluster size for fetal Leydig cells as single (1 cell per cluster), small (2–4 cells per cluster), medium (5–16 cells per cluster), and large (> 16 cells per cluster) cluster. As shown in Figure 5, ATR significantly increased the percentage of medium and large clusters at a dose of 100 mg/kg. These data suggest that ATR causes abnormal aggregation of fetal Leydig cells at the high dose.

### ATR Does Not Alter Sertoli Cell Number

Fetal Sertoli cells were stained with its biomarker SOX9 (Supplementary Figure S2). ATR did not alter fetal Sertoli cell number either. This suggests that fetal Sertoli cell proliferation is not influenced.

### ATR Regulates Testicular Cell Gene Expression

**FIGURE 6:**
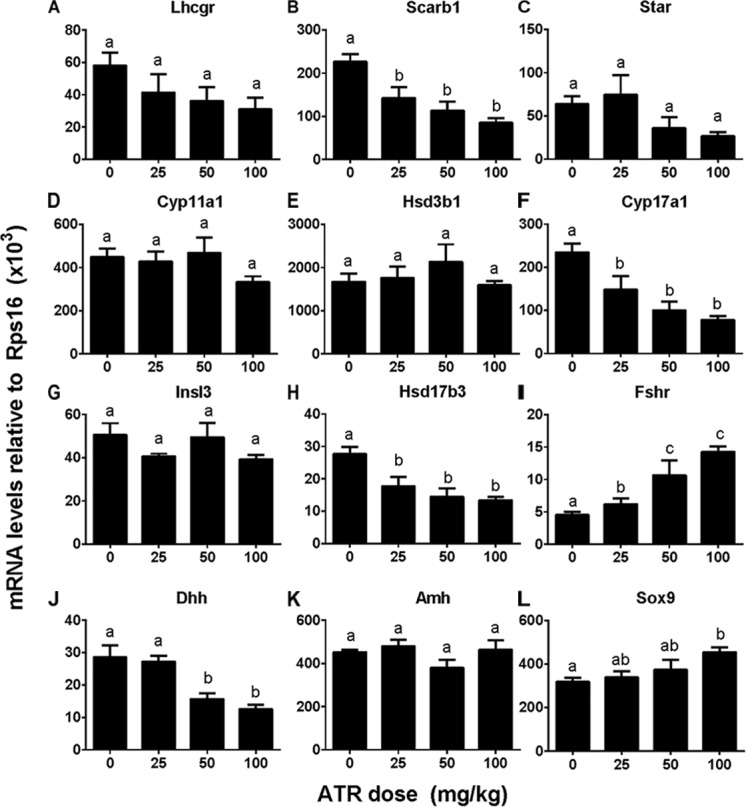
Gene expression levels in fetal testis after atrazine exposure. Fetal Leydig cell genes: **(A)** Lhcgr, **(B)** Scarb1, **(C)** Star, **(D)** Cyp11a1, **(E)** Hsd3b1, **(F)** Cyp17a1, **(G)** Insl3. Fetal Sertoli cell genes: **(H)** Hsd17b3, **(I)** Fshr, **(J)** Dhh, **(K)** Amh, and **(L)** Sox9. These data were adjusted by Leydig cell number. Mean ± SE, *n* = 6. Identical letters designate no difference between two groups at *P* < 0.05.

We investigated effects of ATR on gene expression in the testis, including fetal Leydig cell genes (*Lhcgr, Scarb1, Star, Cyp11a1, Hsd3b1, Cyp17a1*, and *Insl3*) and Sertoli cell genes (*Hsd17b3, Fshr, Dhh, Amh*, and *Sox9*). Without adjustment of cell number, ATR up-regulated *Cyp11a1, Hsd3b1, Insl3, Fshr*, and *Sox9* expression at 50 and/or 100 mg/kg and down-regulated *Hsd17b3* and *Dhh* expression at 25–100 mg/kg without affecting others (Supplementary Figure S3). After adjustment of fetal Leydig and Sertoli cell number, ATR up-regulated *Fshr* and *Sox9* expression and down-regulated *Scarb1, Cyp17a1, Hsd17b3*, and *Dhh* expression without affecting others (Figure 6). The mRNA levels after adjustment of fetal Leydig and Sertoli cell number suggest that ATR alters fetal Leydig and Sertoli cell gene expression.

### ATR Affects Testis Protein Levels

**FIGURE 7:**
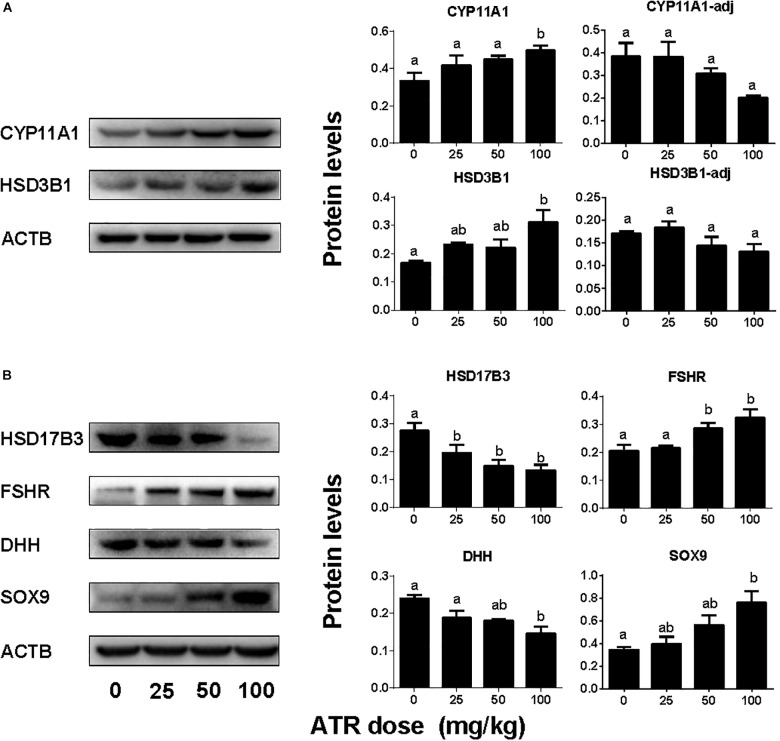
Protein expression levels of fetal Leydig and Sertoli cells. Panel **(A)** Leydig cell proteins; Panel **(B)** Sertoli cell proteins. Leydig cell protein levels include original data (CYP11A1 and HSD3B1) and the levels adjusted to Leydig cell number (CYP11A1-adj) and (HSD3B1-adj). Mean ± SE, *n* = 6. Identical letters designate no difference between two groups at *P* < 0.05.

**FIGURE 8:**
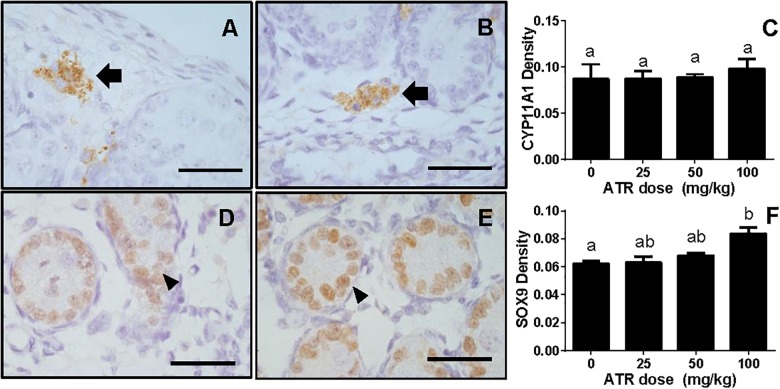
Semi-quantitative measurement of CYP11A1 in the fetal Leydig cell and SOX9 in the fetal Sertoli cell *per se*. Panel **(A,B)**, representative images of CYP11A1 at 0 and 100 mg/kg ATR, respectively; Panel **(D,E)**, representative images of SOX9 at 0 and 100 mg/kg ATR, respectively. Arrow points to CYP11A1-staining in the Leydig cell; Arrowhead points to SOX9 staining in the Sertoli cell. Bar = 50 μm. Panel **(C,F)**, quantitation of CYP11A1 and SOX9 levels. Mean ± SE, *n* = 6. Identical letters designate no difference between two groups at *P* < 0.05.

The levels of Leydig cell proteins (CYP11A1 and HSD3B1), and Sertoli cell proteins (HSD17B3, FSHR, DHH, and SOX9) were measured and analyzed by Western blotting analysis and quantitated (Figure 7) and CYP11A1 and SOX9 protein levels per cell were also further analyzed by semi-quantitative immunohistochemical staining (Figure 8). These results suggest that the protein levels of several androgen synthetic enzymes are altered, which confirm their mRNA levels. We speculate that the change in protein levels is partly due to mRNA regulation, because mRNA carries genetic information that directs protein synthesis. In addition, these results indicate a potential influence of ATR on protein expression.

## Discussion

Here we report that ATR reduces serum testosterone levels at a dose of 100 mg/kg, indicating that ATR can interfere with androgen biosynthesis. ATR induces abnormal aggregation of fetal Leydig cells and increases fetal Leydig cell number via the mitosis at a dose of 100 mg/kg, but does not affect Sertoli cell number. Although the fetal Leydig cell number is increased, the expression of important fetal Leydig cell genes such as *Scarb1* and *Cyp17a1* is down-regulated. Furthermore, the significant down-regulation of *Hsd17b3* in Sertoli cells at doses of 25–100 mg/kg might lead to the decreased secretion of testicular testosterone. The down-regulation of these transcripts might override the effects of the increased fetal Leydig cell number, leading to the lower androgen level.

We found that ATR treatment at doses of 25, 50, and 100 mg/kg/day did not affect the body weight gains of pregnant rats and their pup weights. However, as observed in a previous study, a dose (125 mg/kg) higher than 100 mg/kg/day could significantly lower dam body weight gain (Desesso et al., 2014) and cause post-implantation loss (Desesso et al., 2014). Therefore, the highest dose (100 mg/kg) of ATR was adopted in the current study. This dose of ATR did not cause apparent general toxicity.

Some endocrine disruptors, such as anti-androgenic phthalates (Mahood et al., 2005; Li et al., 2014, 2016) and estrogen-like insecticide methoxychlor metabolites (Liu et al., 2016), can significantly increase the incidence of MNGs after *in utero* exposure. However, ATR did not have such effects (Supplementary Figure S1), suggesting that ATR has a different reproductive toxicity mechanism from the phthalates and methoxychlor.

Interestingly, ATR significantly increased fetal Leydig cell number at a dose of 100 mg/kg (Figure 3). Although the exact mechanism is not clear, apparently ATR increased the fetal Leydig cell mitosis rate as shown by the significant increase of PCNA-labeling index of fetal Leydig cells (Figure 4).

Like some phthalates, ATR also significantly increased the abnormal aggregation of fetal Leydig cells at a dose of 100 mg/kg, leading to a significant increase of medium (5–16 cells per cluster) and large (> 16 cells per cluster) clusters (Figure 5). Additionally, fetal Leydig cells number was also induced by ATR in a dose-dependent manner, thus we speculate that increased fetal Leydig cell numbers could cause its abnormal aggregation. The mechanism of abnormal fetal Leydig cell aggregation is still unclear. Previous studies in phthalate-induced fetal Leydig cell aggregation indicate that this abnormal aggregation of fetal Leydig cells is not related with the decrease of testosterone production in the fetal testis (Mahood et al., 2005; Lin et al., 2008).

Interestingly, the present study clearly demonstrated that gestational exposure to ATR down-regulated the critical enzyme, HSD17B3 in fetal Sertoli cells. In mice, HSD17B3 has been found to be primarily expressed in fetal Sertoli cells (Shima et al., 2013). In rats, when fetal Leydig cells were depleted by a drug ethane dimethane sulfonate, *Hsd17b3* was slightly increased, suggesting that it is also mainly expressed in the seminiferous tubules (Su et al., 2018). However, other androgen biosynthetic enzymes such as CYP11A1, HSD3B1, and CYP17A1 are exclusively present in fetal Leydig cells (Shima et al., 2013; Su et al., 2018). Thus, in fetal Leydig cells, cholesterol can be converted into pregnenolone by CYP11A1 in the mitochondrial inner membrane and pregnenolone can be converted into progesterone and further to androstenedione in the smooth endoplasmic reticulum after HSD3B1 and CYP17A1 catalysis. Androstenedione can diffuse into fetal Sertoli cells, where it is converted to testosterone by HSD17B3. Thus, the down-regulation of mRNA of several androgen-related biosynthetic enzymes such as *Scarb1* and *Cyp17a1* as adjusted by fetal Leydig cell number, *Hsd17b3* and *Dhh* in fetal Leydig and Sertoli cells could affect their corresponding protein expression, which further decrease testosterone secretion as shown in Figure 2.

DHH has been shown to play a critical role in regulating the normal development of fetal Leydig cells (Bitgood et al., 1996; Morales et al., 2009). Knockout of *Dhh* gene in mice showed a significant delay of development of fetal Leydig cells (Yao et al., 2002). Indeed, several critical transcripts in fetal Leydig cells such as *Scarb1* and *Cyp17a1* as judged by the fetal Leydig cell *per se* were significantly down-regulated.

However, the prenatal exposure to ATR up-regulates the expression of *Sox9* and *Fshr* in Sertoli cells. SOX9 is a critical transcription factor for the determination of pre-Sertoli cells into the Sertoli cell lineage. The up-regulation of SOX9 indicates the determination of pre-Sertoli cells was not altered. Indeed, this was supported by the up-regulation of *Fshr*, the Sertoli cell maturation biomarker.

Although the function of fetal Leydig cells is blocked after ATR treatment, the number of fetal Leydig cells is increased. Thus, many transcripts of fetal Leydig cells without adjustment of the Leydig cell number, such as *Cyp11a1, Hsd3b1*, and *Insl3*, were increased. However, after adjustment of the Leydig cell number, their levels were not changed, suggesting that the expression in the fetal Leydig cell *per se* is not altered. The homeostasis of androgen in fetal Leydig cells depends on fetal Leydig and Sertoli cell number, the expression levels of steroidogenesis-related proteins in both fetal Leydig and Sertoli cells *per se*. In this regard, the significant decrease in HSD17B3 could be the major cause of the lower testosterone production after ATR treatment. This decrease of testosterone production confirms a previous observation that ATR at the higher dose led to the reduction of testosterone production in fetal rat testis (Fraites et al., 2011).

A previous study has shown that the median level of a major ATR metabolite diaminochlorotriazine in human urine samples was 0.32 ng/mL (0.16–0.45 ng/mL) (Namulanda et al., 2017), which is far lower than doses used in the current study (25–100 mg/kg). However, most toxicological studies adopted 1–100 mg/kg ATR dose range (Babic-Gojmerac et al., 1989; Friedmann, 2002; Stoker et al., 2002). Therefore, the data collected in the current study imply the toxicological mechanism of atrazine in fetal rats. Therefore, the exact toxicity for human reproduction needs further investigation in the future.

**FIGURE 9:**
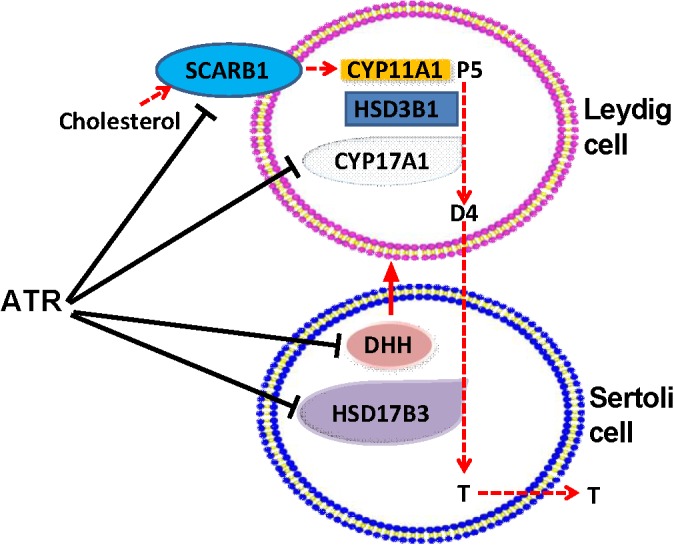
Illustration of the actions of atrazine on Leydig and Sertoli cells. Cholesterol is transported by SCARB1 into fetal Leydig cells, where was used by CYP11A1 to generate pregnenolone (P5), which was further catalyzed by HSD3B1, and CYP17A1 to generate androstenedione (D4). D4 is diffused to Sertoli cells, where HSD17B3 catalyzes it into testosterone (T), which is secreted. ATR lowered SCARB1 and CYP17A1 in Leydig cells and HSD17B3 in Sertoli cells, thus blocking T biosynthesis. ATR also inhibited the expression of Dissert Hedgehog (DHH), which is a critical factor for fetal Leydig cell function.

Collectively, ATR increases fetal Leydig cell number but blocks the function of the fetal Leydig cells via affecting the secretion of DHH in the fetal Sertoli Leydig cells. Together with the down-regulation of HSD17B3 in the fetal Sertoli cells, the delayed maturation of fetal Leydig cells contributes to the lower testosterone production after *in utero* ATR exposure (Figure 9).

## Author Contributions

QL and R-sG conceptualized the study design and analyzed the data. YF, CN, YD, HL, SW, XL, YL, and TH performed the experiments and collected the data. R-sG wrote the manuscript.

## Conflict of Interest Statement

The authors declare that the research was conducted in the absence of any commercial or financial relationships that could be construed as a potential conflict of interest.

## Supplementary Material

FIGURE S1 Occurrence of multinucleated gonocytes (MNGs) after atrazine treatment. Sections were stained with hematoxylin-eosin. The representative photomicrographs of rat testis sections after the treatment of 0 **(A)**, 25 **(B)**, 50 **(C)**, and 100 mg/kg **(D)** ATR. Black arrows point to MNGs in the fetal testis. Scale bar = 50 μm. **(E)** The percentage of MNGs in the fetal testis. Identical letters designate no difference between two groups at P < 0.05.

FIGURE S2 Effects of atrazine of Sertoli cell number. Sections were stained immunohistochemically for SOX9. Quantitative data of fetal Sertoli cell number was calculated. Mean ± SE, *n* = 6. Identical letters designate no difference between two groups at *P* < 0.05.

FIGURE S3 Gene expression levels in fetal testis after atrazine exposure without adjustment of fetal Leydig and Sertoli cell numbers. Fetal Leydig cell genes: **(A)** *Lhcgr*, **(B)** *Scarb1*, **(C)** *Star*, **(D)** *Cyp11a1*, **(E)** *Hsd3b1*, **(F)** *Cyp17a1*, **(G)** *Insl3*. Fetal Sertoli cell genes: **(H)** *Hsd17b3*, **(I)** *Fshr*, **(J)** *Dhh*, **(K)** *Amh*, and **(L)** *Sox9*. Mean ± SE, *n* = 6. ^∗^*P* < 0.05, ^∗∗^*P* < 0.01, ^∗∗∗^*P* < 0.001.

TABLE S1 Antibodies.

TABLE S2 Primer information.
