# Supplementary figures and images for: In utero Exposure to Atrazine Disrupts Rat Fetal Testis Development
Source: Front Pharmacol. 2018 Nov 28;9:1391. doi: 10.3389/fphar.2018.01391 (PMC6280720; doi:10.3389/fphar.2018.01391)

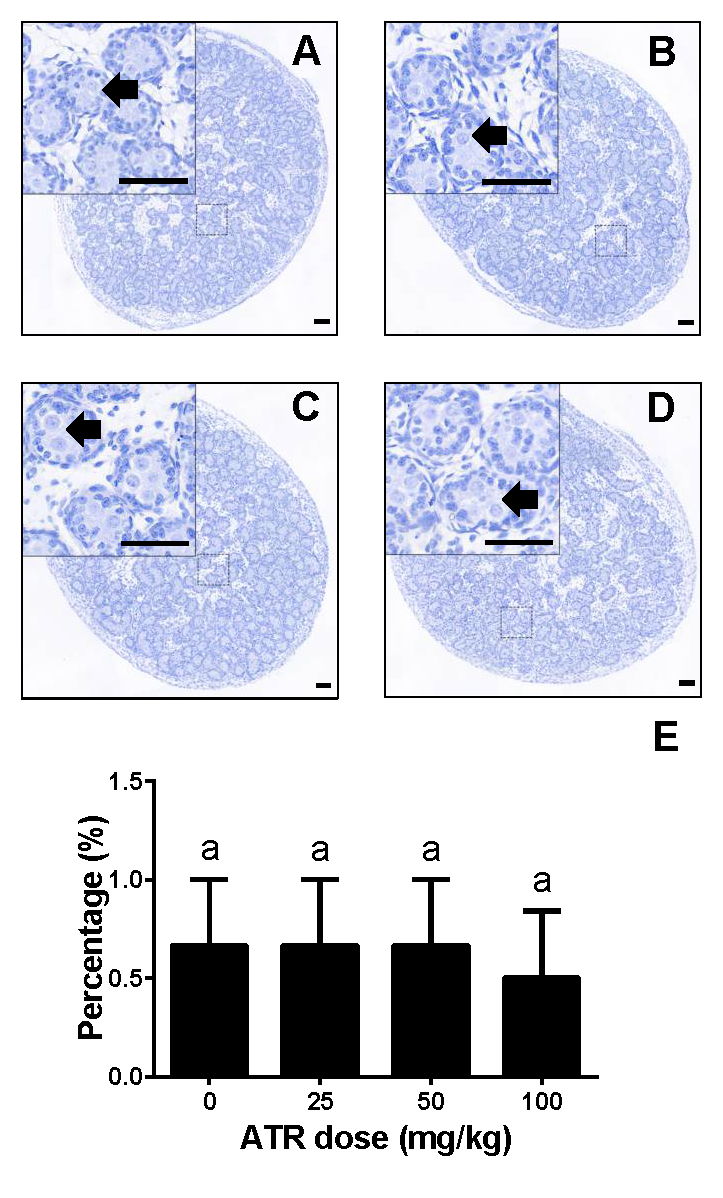

Supplement: FIGURE S1 — Occurrence of multinucleated gonocytes (MNGs) after atrazine treatment. Sections were stained with hematoxylin-eosin. The representative photomicrographs of rat testis sections after the treatment of 0 (A), 25 (B), 50 (C), and 100 mg/kg (D) ATR. Black arrows point to MNGs in the fetal testis. Scale bar = 50 μm. (E) The percentage of MNGs in the fetal testis. Identical letters designate no difference between two groups at P < 0.05. [file Image_1.TIFF]
