# Supplementary material for: In utero Exposure to Atrazine Disrupts Rat Fetal Testis Development
Source: Front Pharmacol. 2018 Nov 28;9:1391. doi: 10.3389/fphar.2018.01391 (PMC6280720; doi:10.3389/fphar.2018.01391)
Supplement: TABLE S1 — Antibodies. [file Table_1.docx]

**Supplementary Table S1. Antibodies**

| **Antibody** | **Species** | **Vendor (City, State, catalogue)** | **Dilution** | |
| --- | --- | --- | --- | --- |
|  |  |  | **WB** | **HS** |
| CYP11A1 | rabbit | Cell Signaling Technology (Danvers, MA) | 1:1000 | 1:500 |
| HSD3B1 | mouse | Abcam (San Francisco, CA) | 1:1000 | ND |
| HSD17B3 | rabbit | Abcam (San Francisco, CA) | 1:2000 | ND |
| FSHR | rabbit | Abcam (San Francisco, CA) | 1:1000 | ND |
| DHH | mouse | Santa Cruz (Santa Cruz, CA) | 1:1000 | ND |
| SOX9 | rabbit | Abcam (San Francisco, CA) | 1:1000 | 1:1000 |
| PCNA | mouse | Abcam (San Francisco, CA) | ND | 1:500 |
| ACTB | rabbit | Cell Signaling Technology (Danvers, MA) | 1:1000 | ND |
|  |  |  |  |  |

ND = Not detected; WB = Western blot; HS = Histochemical staining.
