# Supplementary material for: In utero Exposure to Atrazine Disrupts Rat Fetal Testis Development
Source: Front Pharmacol. 2018 Nov 28;9:1391. doi: 10.3389/fphar.2018.01391 (PMC6280720; doi:10.3389/fphar.2018.01391)
Supplement: Supplementary file 6 [file Data_Sheet_1.docx]

## Supplementary Method

### 2.1 Sample treatment

Dams were euthanized by CO^2^ at GD 21 and body weights of dams and male fetuses were measured. Blood of male fetus was collected and serum was gathered after centrifugation. Sera from male fetuses of the same dam were pooled together. One set of fetal testes (at least one testis per male fetus per pregnant rat) was randomly selected and frozen in liquid nitrogen and stored at -80 ^o^C for subsequent real-time quantitative PCR (qPCR) analysis of fetal testis mRNA levels and Western blot analysis of some selected testis protein levels. Another set of testes was randomly selected and fixed by immersion in Bouin’s solution for 1 day for histochemical, immunohistochemical, and immunofluorescent stainings.

### 2.2 Measurement of serum testosterone level

Serum testosterone level was measured by immunochemiluminometric assay, according to the manufacturer’s instructions using the IMMULITE® 2000 Immunoassay System and Total Testosterone Kit from Siemens Healthcare Diagnostics Products Limited (Gwynedd, UK). The minimal detection limit of testosterone was 0.2 ng/ml.

### 2.3 Histochemical hematoxylin and eosin (HE) staining

Some endocrine disruptors can induce the incidence of multinucleated gonocytes in the fetal testis ([Wen et al., 2014](#_ENREF_33)). Bouin’s solution fixed testes were arrayed in a tissue array in one tissue block. Six-m-thick cross sections were cut and stained with hematoxylin and eosin staining solution. Images of cross sections of fetal testis were taken under a BX53 Olympus microscope. One complete testis cross-section each male fetal rat was analyzed and the percentage of seminiferous cord cross-sections containing multinucleated gonocytes was recorded and calculated as the percentage of the total tubules counted.

### 2.4 Immunohistochemical staining and cell counting

In the current study, CYP11A1 was used as the biomarker of fetal Leydig cells ([Guo et al., 2013](#_ENREF_14)) and SOX9 as the biomarker of fetal Sertoli cells ([Koopman, 1999](#_ENREF_19)). The numbers of CYP11A1 positive fetal Leydig cells and SOX9 positive fetal Sertoli cells were counted using a fractionator technique as previously described ([Mendis-Handagama et al., 1989](#_ENREF_23)). Briefly, six testes per group were randomly collected, embedded in paraffin in a tissue array. Six-m-thick sections from the testis paraffin block were cut. Approximately, ten sections were randomly selected from each testis per rat. Testis sections were subjected to immunohistochemical staining of CYP11A1 or SOX9. Avidin-biotin immunohistochemical staining method was used using a Vector kit (Burlingame, CA) as previously described ([Wu et al., 2017](#_ENREF_34)). Antigen retrieval was performed by heating sections in a citrate buffer (10 mM, pH 6.0) in a heating block for 10 min. 0.5% H_2_O_2_ in methanol was adopted to block the endogenous peroxidase. Sections were incubated with either CYP11A1 or SOX9 antibody (1:1000 dilution, v/v) at room temperature for 1 h. The antibody-antigen complexes were visualized with diaminobenzidine. A cell with a brown mitochondrial staining was designated as a fetal Leydig cell. A cell with a brown nuclear staining was designated as a fetal Sertoli cell. The sections were counterstained with Mayer hematoxylin, dehydrated in graded concentrations of ethanol. Sections in the glass were covered with resin. Images were taken using a mosaic microscope and merged for the whole testis section. The total number of fetal Leydig or Sertoli cells were calculated using a stereological method as described ([Mendis-Handagama et al., 1989](#_ENREF_23)).

### 2.5 Quantitative immunohistochemical measurement of CYP11A1 and SOX9

The level of a protein in the tissue not only depends on its expression level but also on the cell number. The levels of CYP11A1 per fetal Leydig cell and SOX9 per fetal Sertoli cell were measured using quantitative immunohistochemical staining. Staining of CYP11A1 and SOX9 was performed as stated above. The density of the target protein and background area was measured using the Image-Pro 6 Plus analysis software according to the manufacturer’s instruction. Fifty fetal Leydig or Sertoli cells per testis were calculated and averaged as previously described ([Liu et al., 2016](#_ENREF_22)).

### 2.6 Measurement of Leydig cell proliferation

The proliferation of fetal Leydig cells was judged by immunofluorescent staining of PCNA after the dual staining of PCNA (proliferating cell) and CYP11A1 (fetal Leydig cell) in the testis. The sections in the tissue array assembled above were used. Sections were incubated with the primary antibodies of CYP11A1 and PCNA sequentially for 60 min. The fluorescent secondary antibody (Alexa-conjugated anti-rabbit or anti-mouse IgG, 1:500) was used to label fetal Leydig cell (CYP11A1, mitochondrial staining in green color) and proliferating cell (PCNA, nuclear staining in red color). Images were taken with a fluorescent microscopy and merged.

### 2.7 Real-time quantitative PCR (qPCR)

To determine Leydig cell specific gene expression levels, qPCR was used. Total RNA was extracted from a frozen testis by a TRIzol kit according to the manufacturer’s instruction (Invitrogen, USA). First strand cDNA synthesis and qPCR were performed as previously described ([Lin et al., 2008a](#_ENREF_20)). Ribosomal protein S16 (Rps16) mRNA level was assayed in each sample as the internal control as previously described ([Lin et al., 2008a](#_ENREF_20)). Primers for testicular genes of interest are listed in supplementary Table S2, including the membrane receptor gene luteinizing hormone receptor (Lhcgr), cholesterol transporting genes, e.g., cholesterol HDL receptor (Scarb1) and steroidogenic acute regulatory protein (Star), and steroidogenic enzyme genes, CYP11A1 (Cyp11a1), HSD3B1 (Hsd3b1), CYP17A1 (Cyp17a1), HSD17B3 (Hsd17b3), as well as other genes INSL3 (Insl3), FSHR (Fshr), DHH (Dhh), AMH (Amh), and SOX9 (Sox9). The relative mRNA levels of the target genes were normalized to Rps16 (internal control) using a standard curve method as previously described ([Ge et al., 2005](#_ENREF_13)). The gene names, symbols, and functions are as described in supplementary Table S2.

### 2.8 Western blotting

Fetal testes were homogenized and then lysed using a radio-immunoprecipitation assay (RIPA) and PMSF (RIPA: PMSF=100:1) buffer (Bocai Biotechnology, China). Total protein concentrations were determined using the BCA assay kit according to the manufacturer’s instruction (Galen Biopharm, Beijing, China). Protein in the amount of 30 g for each sample was loaded and electrophoresed using a SDS–PAGE gel (10% w/v acrylamide) and then blotted onto a polyvinylidene fluoride membrane (Bio-Rad, Hercules, CA). Nonspecific bindings were blocked with nonfat milk powder (5% w/v) in a tris-buffered saline tween-20 buffer (TBST) for 1 h. After that, the membranes were incubated at 4 ^o^C overnight with primary antibodies against the antigens (listed in the supplementary Table S1). The membranes were then washed and incubated with HRP-conjugated anti-rabbit or anti-goat IgG secondary antibody (1:2000, Abcam, San Francisco, CA) for 2 h at room temperature and washed 3 times. Blots were stripped and incubated with a polyclonal-actin (ACTB) antibody served as the internal control. The band was visualized and the density was calculated using J-Software.
